# (Zn,H)-codoped copper oxide nanoparticles via pulsed laser ablation on Cu-Zn alloy in water

**DOI:** 10.1186/1556-276X-7-272

**Published:** 2012-05-30

**Authors:** Bo-Cheng Lin, Shuei-Yuan Chen, Pouyan Shen

**Affiliations:** 1 Department of Materials and Optoelectronic Science, National Sun Yat-sen University, Kaohsiung, 80424, Taiwan; 2 Department of Mechanical and Automation Engineering, I-Shou University, Kaohsiung, 84001, Taiwan

**Keywords:** copper oxide, nanocondensate, (Zn,H) codopant, PLA, water

## Abstract

Nanosized (5 to 10 nm) amorphous and crystalline nanocondensates, i.e., metallic α-phase of Zn-Cu alloy in face-centered cubic structure and (Zn,H)-codoped cuprite (Cu_2_O) with high-pressure-favored close-packed sublattice, were formed by pulsed laser ablation on bulk Cu_65_Zn_35_ in water and characterized by X-ray/electron diffractions and optical spectroscopy. The as-fabricated hybrid nanocondensates are darkish and showed photoluminescence in the whole visible region. Further dwelling of such nanocondensates in water caused progressive formation of a rice-like assembly of (Zn,H)-codoped tenorite (CuO) nanoparticles with (001), (100), and {111} preferred orientations, (111) tilt boundary, yellowish color, and minimum bandgap narrowing down to *ca.* 2.7 eV for potential photocatalytic applications.

## Background

Monoclinic tenorite (CuO, space group C2/c) and cubic cuprite (Cu_2_O, space group Pn3m) with the copper atoms on face-centered cubic (fcc) and oxygen atoms on bcc sublattice [1] are the p-type semiconductors having a bandgap of 1.4 and 2.17 eV, respectively [2]. The two copper oxide types are typically formed by static thermal oxidation or chemical reaction processes such as heating Cu under O_2_ environment at high temperatures [2,3], through the Cu(OH)_2_ intermediate at high temperatures [4-7], and via a solvent/stabilizer-specific synthesis for the desired shape of the nanoparticles [8,9]. CuO nanoparticles with unusual rice-shaped architectures were fabricated by specific solvent reaction and oxidation under low temperatures [8], whereas the polyvinyl pyrrolidone (PVP) stabilizer concentration, reaction time, and temperature were found to be responsible for the shape-controlled synthesis of Cu_2_O crystals [9]. On the other hand, a dynamic physical-chemical process such as pulsed laser ablation (PLA) on Cu target in air was also found to form Cu_2_O, CuO, and Cu(OH)_2_ films under the influence of water vapor [10].

Here, PLA on Cu-Zn alloy rather than on pure Cu in water was used to fabricate (Zn,H)-codoped copper oxide nanoparticles and to study the phase behavior as well as optical property change of the colloidal solution upon dwelling in water. We focused on (1) competitive oxidation of Cu vs. Zn against the standard Galvanic cell having copper as the cathode and zinc as the anode, and hence phase selection in the dynamic PLA process in water; (2) (Zn,H) signature and optical properties of the as-fabricated copper oxides in amorphous state and high-pressure-favored cuprite structure; and (3) water dwelling-induced tenorite which assembled as rice-like domains with (Zn,H) signature, preferred orientations, planar defect, and yellowish color indicating a significant bandgap narrowing for potential photocatalytic applications.

## Methods

### PLA process in water

The brass target with a bulk composition of 65 wt.% Cu and 35 wt.% Zn having a predominant α-phase of Zn-dissolved Cu and rather minor unalloyed Cu (both in fcc structure, *cf.* Additional file 1) was subjected to energetic Nd:YAG laser (1,064 nm in wavelength; beam mode: TEM00; Lotis, Minsk, Belarus) pulse irradiation in water. The upper surface of the target was 5 mm below the water level in a beaker of 6 cm in diameter full of deionized water *ca.* 15 cm^3^ in volume during such a PLA process. Laser beam was focused to a spot size of 0.03 mm^2^ on the target at 800 mJ/pulse for a peak power density of 1.7 × 10^11^ W/cm^2^ (average power density 2.6 × 10^4^ W/cm^2^) given a pulse time duration of 16 ns at 10 Hz under Q-switch mode.

### Characterization

The nanocondensates produced by PLA in the colloidal solution were centrifuged and then collected/deposited on a glass substrate for phase identification by X-ray diffraction (XRD; D1, Cu Kα at 45 kV, 35 mA, and 3 to 5 s for each 0.01° increment from 30 to 60 of 2*θ* angle; Siemens, Munich, Germany). The same deposit was studied by X-ray photoelectron spectroscopy (XPS; JPS-9010MX photoelectron spectrometer with Mg KR X-ray source; JEOL, Akishima-shi, Japan) calibrated with a standard of C 1*s* at 284.2 eV regarding the Zn 2*p*3/2 peak, Cu, and O 1*s*. The composition and crystal structures of the individual condensates collected on nickel grids overlaid with a carbon-coated collodion film were characterized by field emission transmission electron microscopy (TEM; Tecnai G2 F20 at 200 kV; FEI, Hillsboro, OR, USA) coupled with selected area electron diffraction (SAED), and point-count energy-dispersive X-ray (EDX) analysis at a beam size of 5 nm.

The UV-visible absorption of the colloidal solution as formed by PLA and that after prolonged dwelling in water at room temperature was characterized using the instrument U-3900 H (Hitachi, Chiyoda-ku, Japan) with a resolution of 0.1 nm in the range of 200 to 900 nm. The powdery condensates as formed by PLA and those after prolonged dwelling in water were used to acquire room-temperature photoluminescence (under 325-nm excitation using a He-Cd lamp laser as the excitation source) and Raman spectrum using a semiconductor excitation laser (633 nm) having a spatial resolution of 1 μm (HR800, Horiba, Kyoto, Japan). The same powdery samples were also mixed with KBr for Fourier transform infrared spectroscopy (FTIR; 66v/S, 64 scans with 4 cm^−1^ resolution; Bruker, Madison, WI, USA) study of the OH^−^ signature.

## Results

### X-ray diffraction

**Figure 1:**
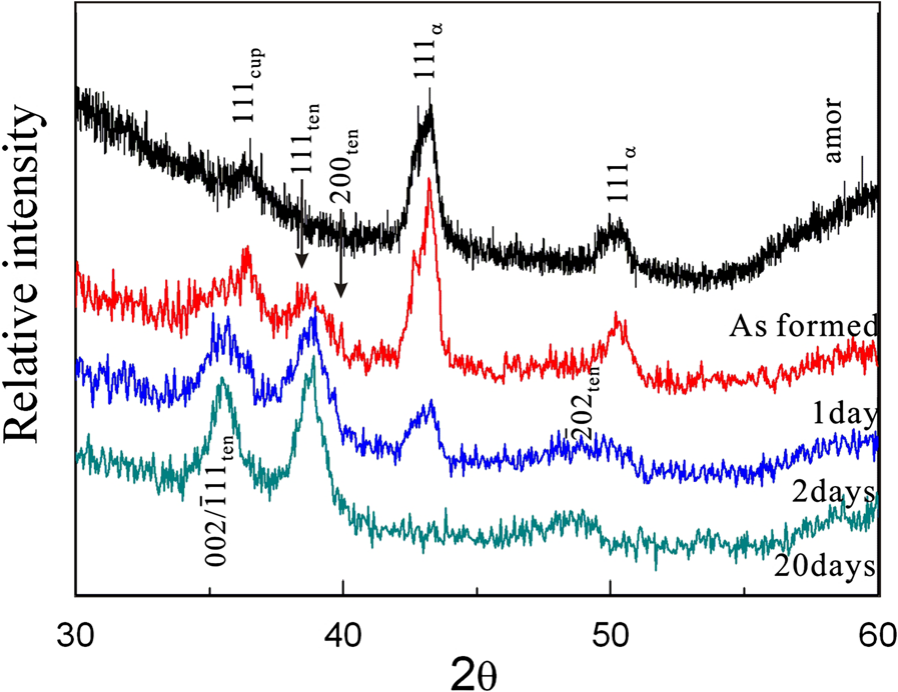
**XRD of the samples as-formed by PLA and further dwelled in water for days.** The specific phases formed are Cu with varied extent of Zn in solid solution (denoted as α), amorphous phase (amor), cuprite (cup), and tenorite (ten) co-doped with Zn^2+^ and H^+^ (*cf.* text).

XRD indicated that the crystalline condensates as prepared by PLA on the Cu_65_Zn_35_ target in water are mainly Cu-Zn solid solution in fcc structure (hereafter, referred to as metallic α-phase) and minor cuprite (Cu_2_O) with significant diffraction broadening (Figure 1). There are also abundant amorphous condensates as indicated by broad diffraction beyond 55° 2*θ* (Figure 1). As compiled in Table 1, further dwelling in water at room temperature for 1 up to 20 days caused progressive formation of tenorite at the expanse of the amorphous phase and the metallic α-phase more than the (Zn,H)-codoped cuprite. In fact, more than 1 day was required to have (Zn,H)-codoped cuprite almost completely transformed into tenorite, and the metallic α-phase almost disappeared after 20 days of dwelling in water. It should be noted that the copper oxides, i.e., cuprite as formed by PLA and tenorite formed subsequently by aging, are (Zn,H)-codoped according to later EDX and FTIR analyses. To our surprise, no Cu(OH)_2_ or Zn(OH)_2_ was detected in the as-formed and further dwelled samples. This is in drastic contrast to our previous observation of the W-ZnO and ϵ-Zn(OH)_2_ composite nanocondensates fabricated by PLA under the same peak power density on Zn target in water [11].

**Table 1:** Specifications of the nanoparticles fabricated by PLA on Cu-Zn alloy in water with optional aging

Sample	**Phase content**	**Size (nm)**	**Shape**
As formed by PLAL	Metallic α	100-500; 5-10	Spherical α particulate; equi-axed nanoparticles
	~ Amor	5 to10	
	>Cu_2_O	5 to10	
	>>W-ZnO	5-10	
Aged for 1 day in water	Metallic α	As above	As above
	>>Amor		
	~ Cu_2_O		
	>>W-ZnO		
Aged for 2 days in water	CuO	5-10 CuO	As above
	> Metallic α		
	>>Cu_2_O	100-300 α	
	>>W-ZnO		
Aged for 20 days in water	CuO	5-10 CuO	Equi-axed nanoparticles assembled as rice-like
	>>W-ZnO		

Metallic α-phase denotes fcc Cu doped with less than 6 wt.% Zn according to EDX analysis; amor denotes an amorphous phase; Cu_2_O (cuprite) and CuO (tenorite) are (Zn,H)-codoped according to EDX and FTIR analyses; W-ZnO was induced mainly by electron irradiation (*cf.* text).

### X-ray photoelectron spectroscopy

**Figure 2:**
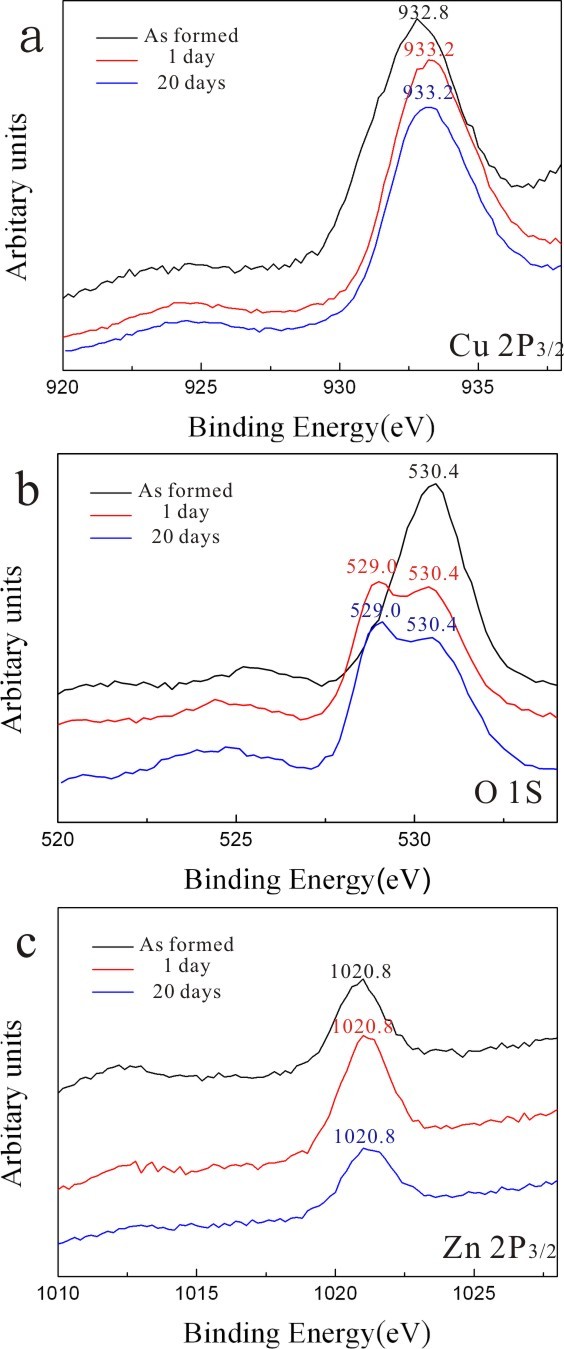
**XPS of the samples as-formed by PLA and further dwelled in water for days.** XPS of the as-formed PLA sample (upper black trace) and the samples with further dwelling in water for 1 (middle red trace) and 20 days (lower blue trace) showing the binding energy change of (**a**) Cu 2*p* from 932.8 eV for Cu^+^ to 933.2 eV for Cu^2+^, (**b**) corresponding shift of O 1*s* from 530.4 to 529.0 eV, and (**c**) relatively inert Zn 2*p* of Zn^2+^ at 1,020.8 eV (*cf.* text).

XPS results of the condensates as prepared by PLA and those with further dwelling in water for 1 and 20 days are compiled in Figure 2. The binding energy of Cu 2*p* turned out to be 932.8 eV for Cu^+^ in the as-formed sample and 933.2 eV for Cu^2+^ in the sample subjected to further dwelling in water (Figure 2a) following the assignments of Poulston et al. [12]. This indicates that the amorphous phase and (Zn,H)-codoped cuprite in the as-formed sample are rich in Cu^+^, whereas the (Zn,H)-codoped tenorite formed later by dwelling in water is rich in Cu^2+^. The corresponding O 1*s* binding energies of O^2−^ are 530.4 and 529.0 eV for Cu^+^- and Cu^2+^-rich phases, respectively (Figure 2b). As for the Zn 2*p* of Zn^2+^ at 1,020.8 eV (Figure 2c), it can be attributed to Zn^2+^ doped in the predominant copper oxides rather than the negligible wurtzite (W)-type ZnO. In fact, this binding energy is lower than the value of pure ZnO (1,022 eV) and remained almost unchanged upon dwelling in water, indicating that the Zn^2+^ state is relatively stable compared to the Cu^+^ and Cu^2+^ states in the present nanocondensates.

### Transmission electron microscopy

**Figure 3:**
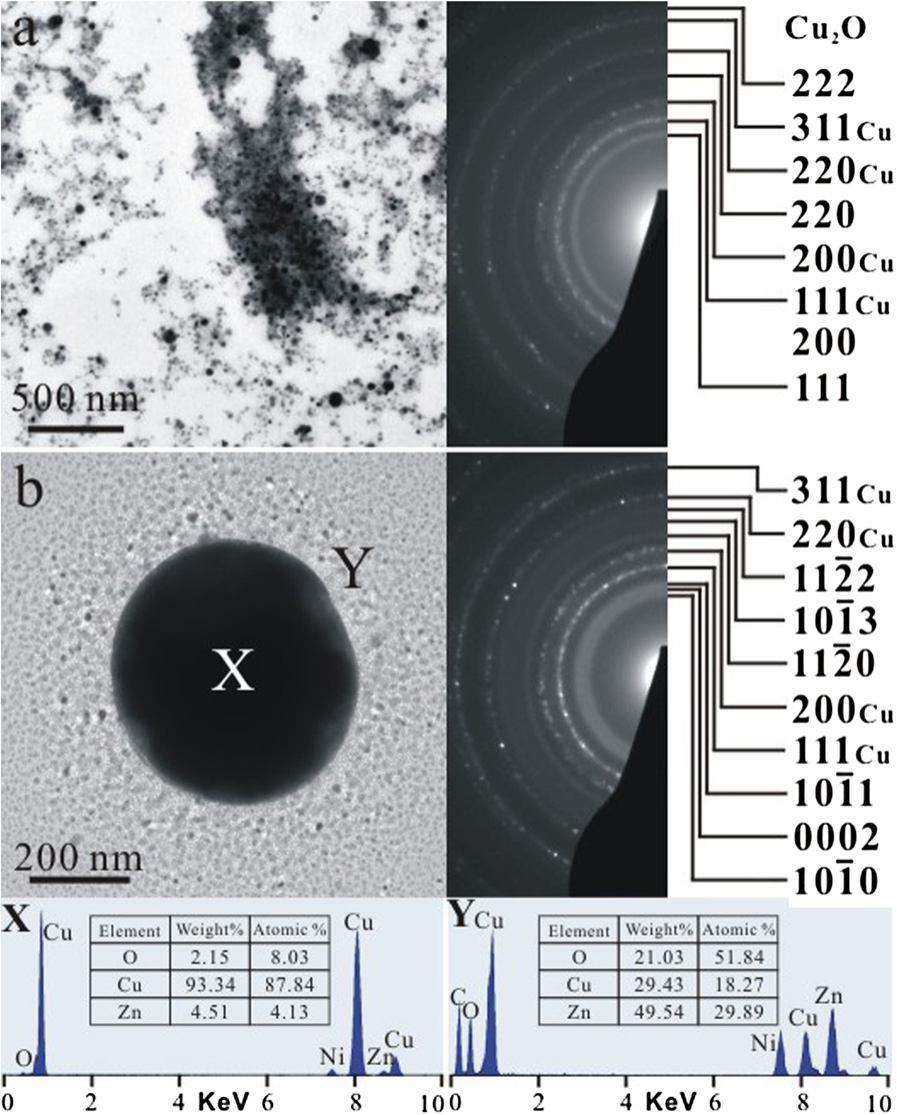
**TEM BFI, SAED, and corresponding EDX of the as-formed PLA sample.** (**a**) Nanocondensates of (Zn,H)-codoped cuprite (Cu_2_O) and metallic α-phase in random orientation and (**b**) submicron-sized Cu particulate slightly dissolved with Zn and co-existing nanoparticles of metallic α-phase and W-ZnO with corresponding point-count EDX analysis at X and Y positions, respectively. Note that W-ZnO in (b) was mainly crystallized from an amorphous matrix upon electron irradiation for 10 min. The Ni counts are from the sample supporting the nickel grid.

**Figure 4:**
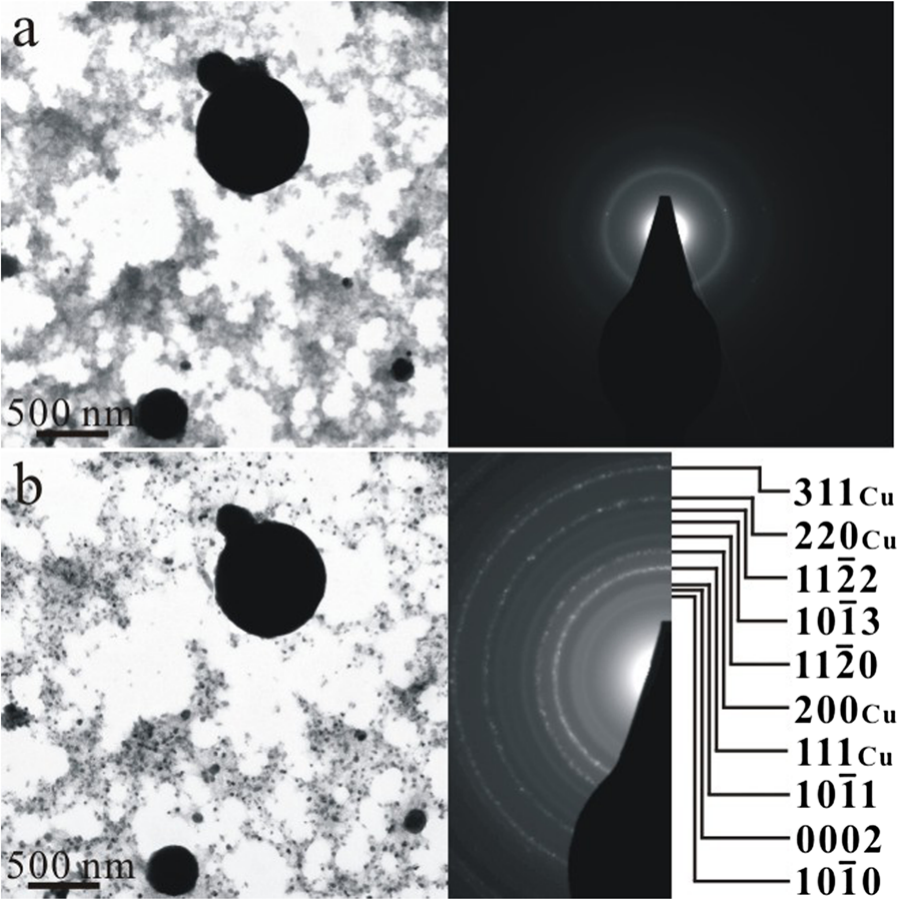
**TEM BFI (left) and corresponding SAED pattern (right).** (**a**) Poorly crystallized condensates prepared by PLA in water and then dwelled in water for 1 day and (**b**) randomly oriented metallic α-phase and W-ZnO nanocrystals newly crystallized from the amorphous phase upon electron irradiation for 20 min.

**Figure 5:**
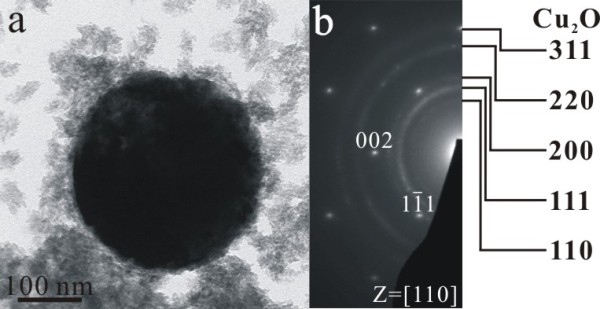
**TEM BFI, SAED, and EDX of cuprite survived in PLA sample aged in water.** TEM (**a**) BFI, (**b**) SAED pattern, and corresponding EDX spectrum of a submicron-sized spherical Cu particle in the [110] zone axis which is surrounded by randomly oriented nanocondensates of (Zn,H)-codoped cuprite (Cu_2_O) which survived dwelling in water for 2 days.

TEM bright field image (BFI) and corresponding SAED pattern of the sample as formed by PLA in water showed nanocrystals of (Zn,H)-codoped cuprite (Cu_2_O) and metallic α-phase *ca.* 5 to 10 nm in size with random orientation (Figure 3a). There are additional submicron-sized (100 to 500 nm) particulates of Cu having only slight Zn in solid solution and amorphous nanocondensates with Zn-Cu-O components as indicated by EDX analysis (Figure 3b). Such amorphous nanocondensates tended to be partially crystallized as W-ZnO and metallic α-phase upon electron irradiation for 20 min (Figure 4). In general, the submicron-sized metallic α-phase particulate surrounded by randomly oriented nanocondensates of (Zn,H)-codoped cuprite (Cu_2_O) survived dwelling in water for 2 days (Figure 5), indicating that the latter were not formed by epitaxial oxidation of the former.

**Figure 6:**
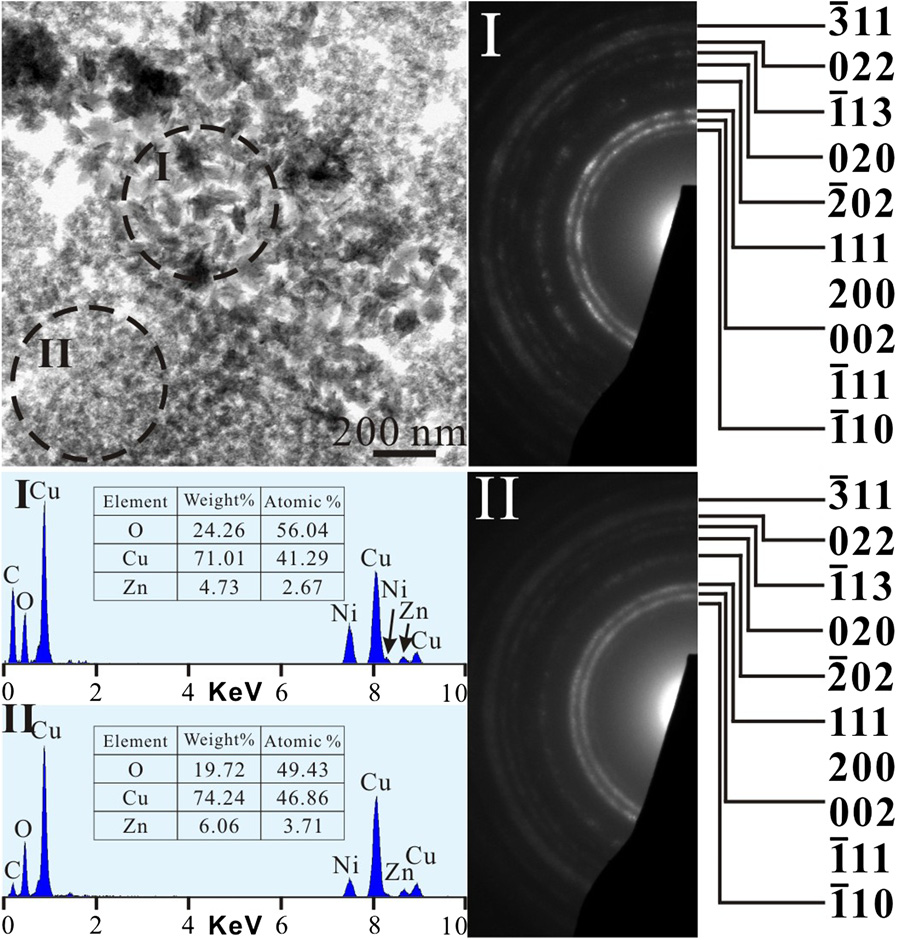
**TEM BFI and SAED pattern of tenorite in PLA sample after prolonged dwelling in water.** Note the slightly preferred orientation of the assembled tenorite nanoparticles in region I to show the (002) and {111} diffraction arcs. The sample was dwelled in water for 20 days after PLA treatment. The Ni counts are from the sample supporting the nickel grid.

**Figure 7:**
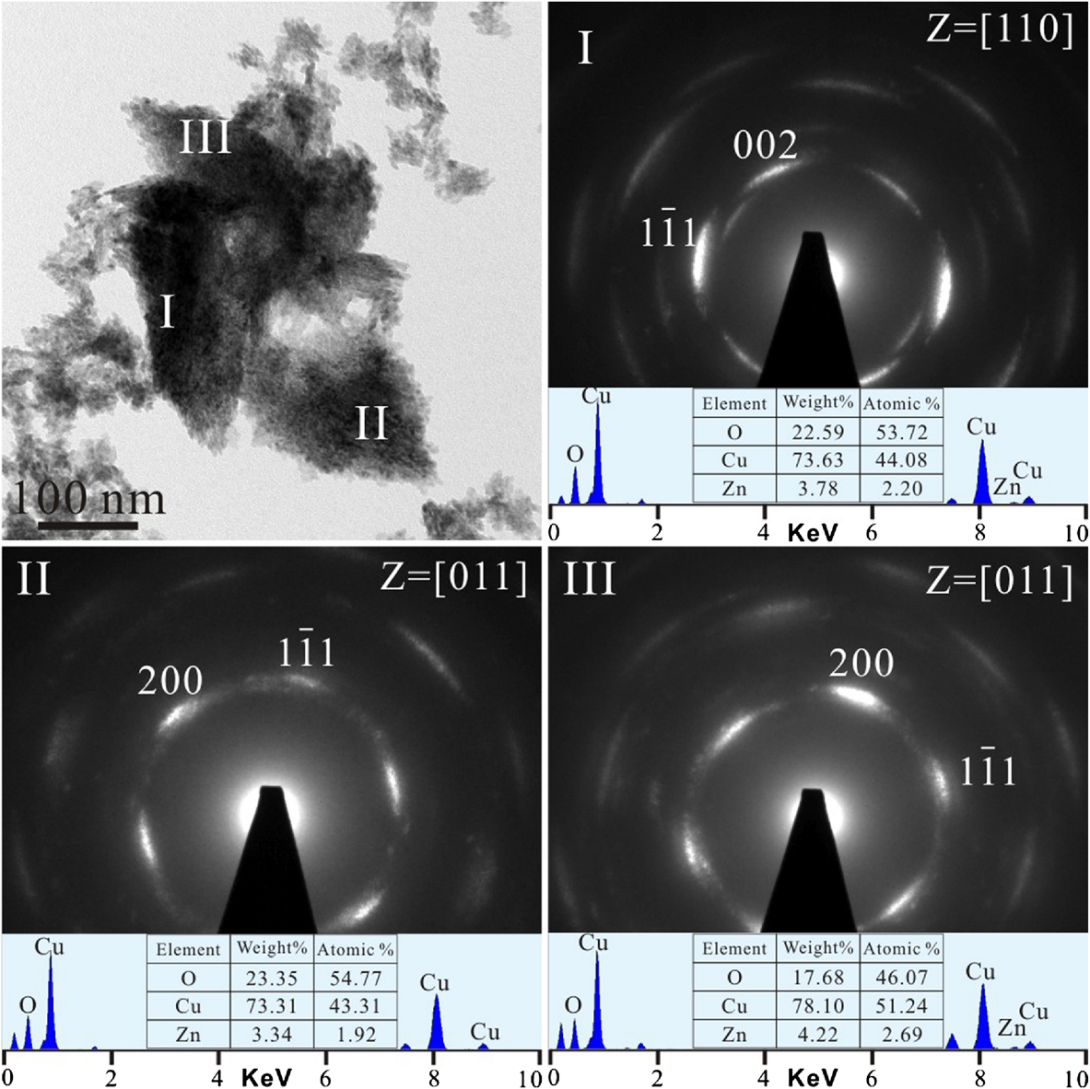
**TEM BFI, SAED, and EDX of preferentially oriented tenorite after prolonged dwelling in water.** The nanoparticles have slight Zn dopant and significant 002, 200, and $1\bar{1}1$ diffraction arcs in the [011] zone axis taken from regions I, II, and III. The same sample as in Figure 6.

**Figure 8:**
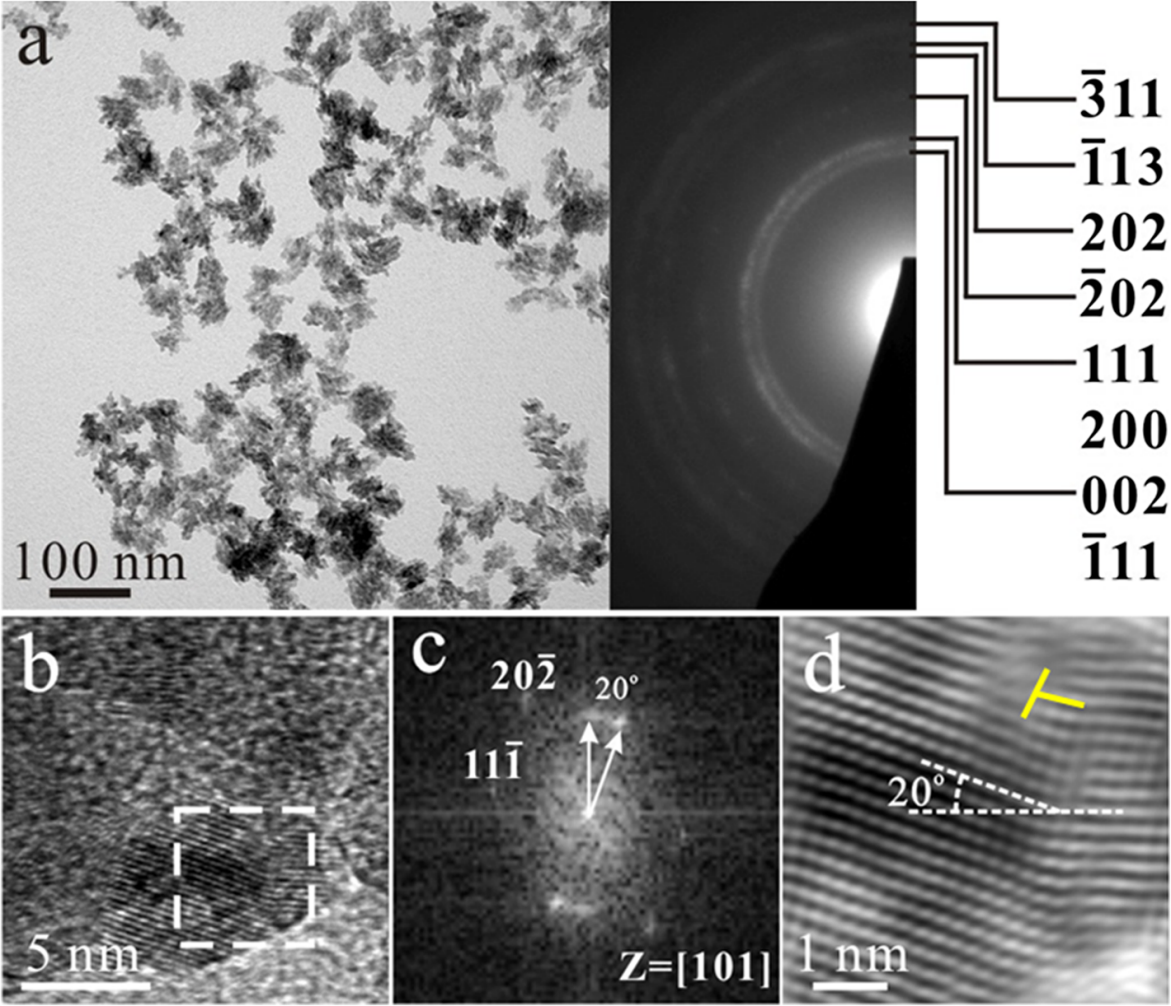
**TEM BFI, SAED, and lattice image of rice-like tenorite nanoclusters with tilt boundary and dislocation.** TEM (**a**) BFI (left) coupled with SAED pattern (right) of the (Zn,H)-codoped tenorite nanocondensate clusters with slightly preferred orientation of (111) plane. (**b**) Lattice image of the individual tenorite nanocondensates *ca.* 5 nm in size which tended to coalesce over (111) to form (111) symmetrical tilt boundary with ($1\bar{1}\bar{1}$) plane off by 20° as indicated by 2-D forward (**c**) and inverse Fourier transform (**d**) of the square region near the [101] zone axis. Note also dislocations (denoted as T) with ($1\bar{1}\bar{1}$) half plane at the interface. The same sample as in Figure 6.

The (Zn,H)-codoped tenorite (CuO) nanoparticles that became predominant after dwelling the PLA sample in water for 20 days showed varied extent of preferred orientation as indicated by the (002), (200), and (111) diffraction arcs (Figures 6, 7, and 8a). The dopant level of Zn also varies from place to place as indicated by point-count EDX analysis. Lattice image (Figure 8b) coupled with two-dimensional (2-D) forward and inverse Fourier transform (Figure 8c,d) indicated that the (Zn,H)-codoped tenorite nanoparticles tended to coalesce over a well-developed (111) surface to form rice-like clusters with (111) tilt boundary having dislocation half plane parallel to ($1\bar{1}\bar{1}$). No cuprite relic was found in this sample to determine its crystallographic relationship with the (Zn,H)-codoped tenorite.

On the basis of the XRD and TEM observations, the phase, size, and shape evolutions of the nanoparticles fabricated by PLA upon dwelling in water are compiled in Table 1.

### Vibrational and PL spectroscopy

**Figure 9:**
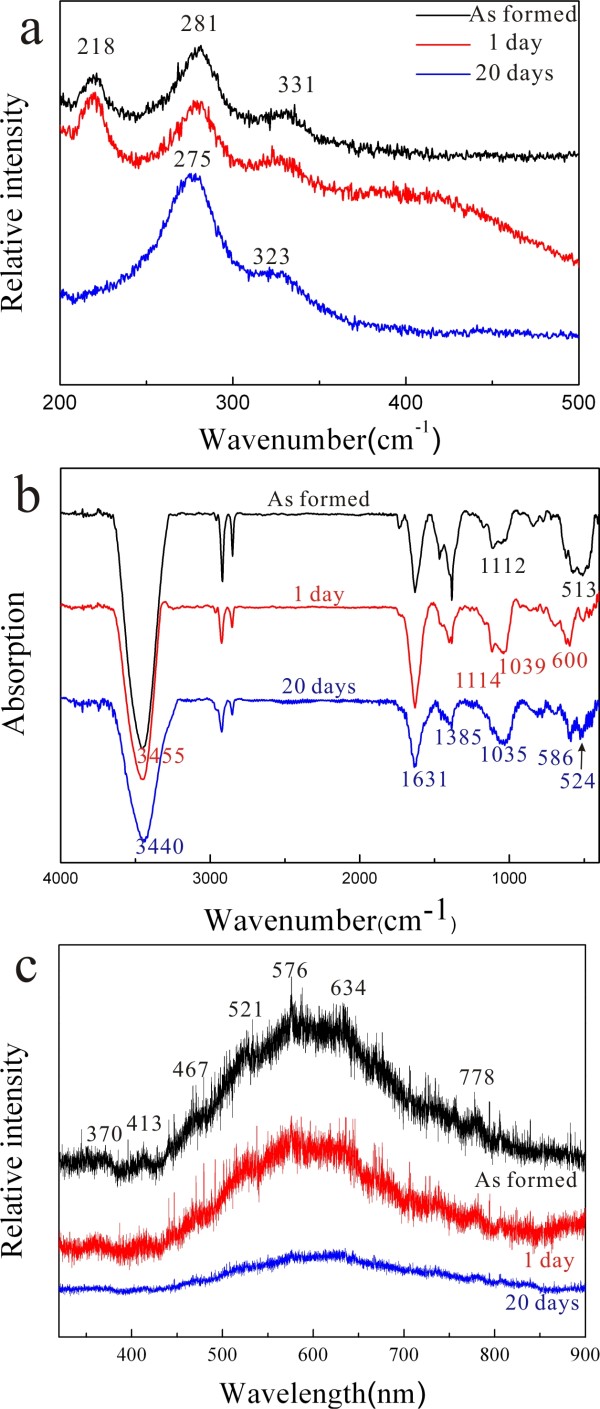
**Raman, FTIR and PL spectra of the (Zn,H)-codoped copper oxide nanocondensates.** (**a**) Raman, (**b**) FTIR, and (**c**) PL spectra of the (Zn,H)-codoped copper oxide nanocondensates prepared by PLA in water (top black trace) followed by dwelling in water for 1 (middle red trace) and 20 days (lower blue trace). The FTIR bands at 2,850 and 2,920 cm^−1^ are due to EtOH contamination during IR sample preparation.

The Raman and FTIR spectra of the (Zn,H)-codoped cuprite (Cu_2_O) and tenorite (CuO) nanocondensates prepared by PLA followed by dwelling in water for 1 and 20 days are compiled in Figure 9. The Raman band at 218 cm^−1^ (Figure 9a) can be assigned as second-order overtone of cuprite according to Sholache-Carranco et al. [13]. The Raman bands at 281 and 331 cm^−1^ can be assigned as A_g_ and B_g_ modes, respectively, for the minor as-formed tenorite which became stronger and shifted to lower wave numbers upon dwelling in water, indicating that new tenorite nanoparticles with finer average size were progressively formed from the colloidal solution. In this regard, the two Raman bands of tenorite (CuO) were reported to shift from 295 to 288 cm^−1^ and 342 to 330 cm^−1^ when the particle size decreases from >100 to 10 nm [14]. It is not clear whether the amorphous phase has similar structure units as the (Zn,H)-codoped cuprite and/or tenorite to affect the observed Raman bands.

The corresponding FTIR spectra (Figure 9b) show significant H signature of the predominant (Zn,H)-codoped cuprite and tenorite in the samples, i.e., OH^−^ band at *ca.* 3,435 to 3,440 nm^−1^ and the band at 1,631 cm^−1^ which can be ascribed to water adsorbed onto the sample surface [15]. The band at 600 cm^−1^ can be assigned to the stretching vibrations (υ Cu(I)-O) of (Zn,H)-codoped Cu_2_O having lower wave number than undoped cuprite (610 cm^−1^) [16]. The bands at 524 and 586 cm^−1^ are due to the stretching vibrations of (Zn,H)-codoped CuO (υ Cu(II)-O) having lower wave number than undoped tenorite (529 and 590 cm^−1^) [16]. There is a slight shift to lower wave number for the condensates upon dwelling in water due to distortion relaxation and/or varied Zn^2+^ and H^+^ signatures. It should be noted that the sample as formed by PLA in water has rather broad band at 513 cm^−1^ presumably due to Zn-O and Cu(I)-O vibration contributions from co-existing cuprite and amorphous phase more or less with (Zn,H) cosignature. As for 1,035 and 1,385 cm^−1^, they could be due to the vibration of (Zn,Cu,H)-cosignified amorphous oxide not previously studied by FTIR. (The bands at 2,850 and 2,920 cm^−1^ are due to EtOH contamination during IR sample preparation.)

The (Zn,H)-codoped copper oxide nanocondensates as formed by the PLA process show a broad photoluminescence (PL) in the whole visible region with multiple peaks at *ca.* 370, 413, 467, 521, 576, 634, and 778 nm (Figure 9c), which can be attributed to various color centers in the (Zn,H)-codoped copper oxides in comparison with a broad emission band centered at 467 nm for CuO [17], two spectral emissions at 388.2 and 753.15 nm for Cu_2_O [18], multiple emissions at 600, 627, and 665 nm for ZnO-CuO composite nanowires [19], green to red (526 to 697 nm) emissions for ZnO-CuO hybrid nanostructures [20], and emissions at 415, 445, 550, and 600 nm for ZnO nanoparticles [21,22]. The PL intensity, however, was progressively weakened with accompanied phase change into a tenorite structure for the nanocondensates after dwelling in water for up to 20 days.

### UV-visible absorption

**Figure 10:**
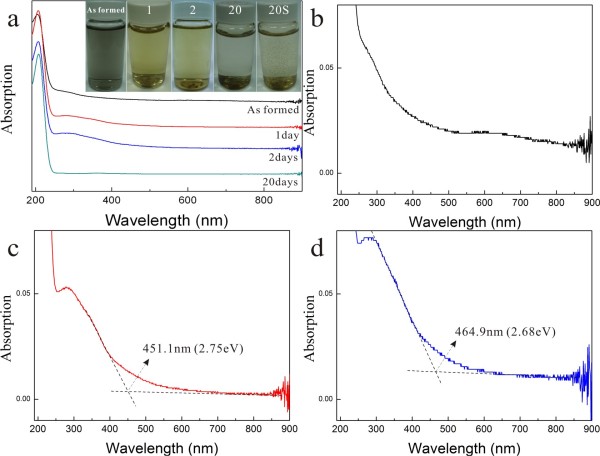
**UV-visible spectra of samples as formed by PLA and further aged in water.** (**a**) UV-visible spectra and color change (inset) of the colloidal solutions as formed by PLA in water and those with further dwelling in water for days numbered. Note the color change from black to light yellow and finally limpid due to sedimentation of significantly coarsened/assembled particles as indicated by the shaken bottle denoted as 20S to the further right of the panel for the solution dwelled in water for 20 days. The magnified spectrum of the as-formed sample in (**b**) shows broad absorbance in the visible region to exhibit a dark color. The samples further dwelled in water for (**c**) 1 and (**d**) 2 days have absorbance corresponding to a minimum bandgap of 2.75 and 2.68 eV based on their intersection with the base line at 451.1 and 464.9 nm, respectively.

The colloidal solution as formed by PLA is dark bluish due to predominant nanoparticles of metallic α-phase besides (Zn,H)-codoped cuprite and amorphous phase, whereas that with further dwelling in water for days is yellowish due to increasing amount of (Zn,H)-codoped tenorite (Figure 10a). The solution finally became limpid due to sedimentation of significantly coarsened/assembled particles which account for little UV-visible absorption of the solution dwelled in water for 20 days. (Note that the sedimentation of the coarsened/assembled particles is indicated by the shaken bottle denoted as 20S in Figure 10a.) The colloidal solution as formed by PLA shows small and broad absorption peaks at 550 to 700 nm (Figure 10b) which could be due to surface plasmon resonance of metallic α-nanoparticles (Cu-Zn solid solution) analogous to the case of Cu nanoparticles embedded in soda-lime glass [23]. In any case, there is broad absorbance in the white region to account for the dark color of the as-formed colloidal solution. By contrast, there is specific absorbance for the sample subjected to further dwelling in water. For example, the absorbance of the samples aged for 1 and 2 days correspond to a minimum bandgap of 2.75 and 2.68 eV based on their intersection with the base line at 451.1 and 464.9 nm, respectively (Figure 10c,d).

## Discussion

### Defect chemistry of (Zn,H)-codoped cuprite and tenorite

The cubic cuprite (Cu_2_O) has Cu^+^ ions in fcc sublattice and oxygen atoms partially filled in the tetrahedral sites [1]. The Zn^2+^ dopant would substitute Cu^+^ with the charge compensated by copper vacancies (V_Cu_'') through the following equation:

(1) $$\text{ZnO}\overset{\text{Cu2O}}{\to }{\text{Zn}}_{\text{Cu}}{}^{\cdot }+\frac{1}{2}{\text{V}}_{\text{Cu}}"+{\text{O}}_{\text{O}}{}^{\text{x}}$$

in the Kröger-Vink notation [24]. Copper divacancies with a trap level at about 0.25 eV may also exist as implied by the deep-level transient spectroscopy study of the heterostructure of *p*-Cu_2_O/*i*-ZnO/*n*-ZnO [25]. The copper vacancies are expected to be richer when hydrogenation introduces more positive changes.

Tenorite (CuO) has Cu^2+^ in a four-coordinate CuO_4_ planar unit [26]. A recent first-principles study showed that CuO is intrinsically a p-type semiconductor because Cu vacancies are the most stable defects in both Cu-rich and O-rich environments [27]. The Zn^2+^ dopant is expected to substitute Cu^2+^ without charge compensation. However, V_Cu_'' are still required to charge compensate the positive charge due to hydrogenation through the following equation:

(2) H2O→CuO2H· + VCu''+OOx.

The various defects by the two equations above and their clusters may account for the observed broad PL emission in the whole visible region for the (Zn,H)-codoped cuprite and tenorite (Figure 9c).

### Phase selection of the Cu-Zn-O-H system via the PLA process

The phase assemblages as formed by the present PLA on Cu-Zn alloy in water are the occasional copper-rich particulates due to rapid cooling through the melting point of pure Cu which increases with increasing pressure *ca.* 2,000 K at 25 GPa [28] and the nanoparticles due to a condensation process. The nanocondensates are mainly the amorphous phase and the crystalline phases having a cubic close-packed structure, i.e., metallic α-phase and cuprite as indicated by XRD results.

Regarding the occurrence of the amorphous phase, pressure-induced amorphization has been reported to occur by static compression of a crystalline material such as silica [29] and by the PLA process for a number of composition systems, such as Al_2_O_3_[30], Cr_2_O_3_[31], and Au [32]. The multiple alloying elements of the present Cu-Zn-O-H system have additional beneficial high entropy for disordering.

As for the cubic close-packed phases, they tended to be stabilized at high temperature and pressure for a lower free energy during the present PLA process in water. The metallic α-phase is, in fact, Zn-doped Cu with fcc structure which is typically favored along with hexagonal close-packed structure at high pressure for many metal elements [33]. The Zn doping to the present Cu condensates, however, prevented them from twinning in drastic contrast to multiple twinned particles of pure Cu clusters *ca.* 5 nm in size by the inert gas aggregation technique [34]. The close-packed cuprite with fcc-based Cu sublattice could be favored at high pressure under the influence of rapid heating and cooling during a typical PLA process [35]. The volume activation could involve (1) intrinsic volume change of the reactants and transition state and (2) volume change associated with solvation effects as of concern also to the mechanisms of inorganic reactions at transition metal sites [36]. By contrast, the monoclinic tenorite was favored upon static dwelling in water under ambient conditions.

It should be noted that W-ZnO was seldom formed unless by electron irradiation on the amorphous nanocondensates (Figure 4). This is surprising because Cu has one and Zn has null unfilled 3*d* electron, so reduction (gain of electrons) would take place for Cu rather than Zn in the solution under standard condition as the case of the Daniell cell, i.e., a Galvanic cell having copper as the cathode and zinc as the anode with a sulfate salt bridge. Such a tendency appears to be invalid in the present PLA on brass in water to form abundant nanoparticles of (Zn,H)-codoped cuprite rather than W-ZnO. It is an open question whether high pressure and/or temperature in the present dynamic PLA process accounts for the reversed oxidation-reduction reaction in the Cu-Zn-O-H system. In any case, the phase assemblage of metallic α-phase, (Zn,H)-codoped cuprite, and amorphous phase is in drastic contrast to the ZnO and CuO hybrid structure formed by directly heating a Cu-Zn alloy [20].

It is also noteworthy that PLA on Cu target in air was reported to form Cu(OH)_2_ besides copper oxides under the influence of water vapor [10]. PLA on Zn target in water was also found to form ϵ-Zn(OH)_2_ besides W-ZnO [11]. By contrast, the present PLA on brass target in water with optional dwelling in water did not cause appreciable Cu(OH)_2_ or Zn(OH)_2_. This can be rationalized by the presence of multiple alloying elements of the present Cu-Zn-O-H system to have additional beneficial high entropy for disordering as mentioned.

### Assembling (Zn,H)-codoped tenorite as rice-like rather than tubular materials

CuO nanoparticles with unusual rice-shaped architectures were fabricated by specific solvent reaction and oxidation under low temperatures [8]. Besides, an interesting shape evolution of Cu_2_O crystals from cubes, truncated octahedra, octahedra, and finally to nanospheres was realized by reducing the copper-citrate complex solution with glucose in low-temperature synthesis [9]. The PVP concentration, reaction time, and reaction temperature are suggested to be responsible for the shape-controlled synthesis of Cu_2_O crystals [9].

The present (Zn,H)-codoped tenorite (CuO) nanoparticles with monoclinic crystal symmetry were also assembled as lenticular- or rice-shaped when the colloidal solution containing the (Zn,H)-codoped cuprite and amorphous phase besides metallic α-phase was dwelled in water for a prolonged time. We suggest that such a rice-like shape is due to (hkl)-specific assembly of the CuO nanoparticles formed at the expense of cuprite and amorphous nanocondensates. The coalescence growth of tenorite (ten) nanoparticles was preferred to occur over the close-packed (001)_ten_ plane with periodic bond chains [37] presumably derived from (111)_cup_ and/or (001)_cup_ of cuprite (cup) in view of the reported crystallographic relationship for copper oxides [38]. Alternatively, tenorite nanoparticles could be nucleated from the (Zn,H)-codoped amorphous phase, i.e., atom clusters via precondensation, to form (200)_ten_ and/or (111)_ten_ surfaces for imperfect coalescence. The (111)-specific coalescence of tenorite, in fact, accounts for the (111) symmetrical tilt boundary (Figure 8) with misfit dislocations at the interface. Such a planar interface would act as an energy cusp to prevent the coalesced nanoparticles from unification upon rapid cooling through the critical temperature for thermally activated Brownian rotation of the particles toward exact epitaxy [39].

## Conclusions

The amorphous phase and close-packed crystalline phase of metallic α-phase and (Zn,H)-codoped cuprite were preferentially formed via the present dynamic PLA on brass in water environment. The formation of copper oxides rather than zinc oxide from metal alloy is reversed from the conventional Galvanic cell having copper as the cathode and zinc as the anode. In addition, the fcc-based cuprite tended to form by the pressure effect under the influence of rapid heating and cooling of the PLA process [35], thus shedding light on the phase behavior of Cu-Zn-O-H system at high temperature and pressure in natural dynamic settings.

As for industrial interest, the (Zn,H)-codoped amorphous phase and cuprite as formed by the PLA process may have potential applications in view of their PL in the whole visible region. Although it is beyond the scope of this study to experimentally explore the photocatalytic reactions of the PLA products, the (Zn,H)-codoped and equi-axed cuprite nanocondensates would be excellent and cheap for photocatalytic reactions such as those involved in the degradation of methyl orange [40], splitting of methanol/water solutions to produce hydrogen [41], and cleaning of organic pollutants from the environment [42]. Besides, the yellowish tenorite nanoparticles with (Zn,H) cosignature and hence minimum bandgap narrowing down to *ca.* 2.7 eV by dwelling in water may have better CO gas sensing efficiency than undoped CuO [19].

## Competing interests

The authors declare that they have no competing interests.

## Authors' contributions

BCL carried out the PLA, XRD, TEM, and vibrational spectroscopic studies; SYC participated in the design and coordination of the study; and PS drafted the manuscript. All authors read and approved the final manuscript.

## Authors' information

BCL is a Ph.D. student at NSYSU. SYC is a professor at I-Shou University. PS is a professor at NSYSU.
